# Latest Insights into Mechanisms behind Atrial Cardiomyopathy: It Is Not always about Ventricular Function

**DOI:** 10.3390/diagnostics11030449

**Published:** 2021-03-05

**Authors:** Bianca-Ana Dmour, Radu-Stefan Miftode, Dan Iliescu Halitchi, Dana Teodora Anton-Paduraru, Codruta-Olimpiada Iliescu Halitchi, Ionela-Larisa Miftode, Ovidiu Mitu, Alexandru-Dan Costache, Celina-Silvia Stafie, Irina Iuliana Costache

**Affiliations:** 1 Department of Internal Medicine I (Internal Medicine), Faculty of Medicine, University of Medicine and Pharmacy “Gr. T. Popa”, 700115 Iasi, Romania; gherasimbianca93@gmail.com; 2 Department of Internal Medicine I (Cardiology), Faculty of Medicine, University of Medicine and Pharmacy “Gr. T. Popa”, 700115 Iasi, Romania; iliescud@gmail.com (D.I.H.); mituovidiu@yahoo.co.uk (O.M.); adcostache@yahoo.com (A.-D.C.); ii.costache@yahoo.com (I.I.C.); 3 Department of Mother and Child Medicine, Faculty of Medicine, University of Medicine and Pharmacy “Gr. T. Popa”, 700115 Iasi, Romania; antondana66@yahoo.com (D.T.A.-P.); codrutzache@yahoo.co.uk (C.-O.I.H.); 4 Department of Infectious Diseases (Internal Medicine II), Faculty of Medicine, University of Medicine and Pharmacy “Gr. T. Popa”, 700115 Iasi, Romania; larisa.miftode@yahoo.com; 5 Department of Preventive Medicine and Interdisciplinarity, Faculty of Medicine, University of Medicine and Pharmacy “Gr. T. Popa”, 700115 Iasi, Romania; celina.stafie@umfiasi.ro

**Keywords:** atrial cardiomyopathy, atrial remodeling, biomarker, fibrosis, cardiac magnetic resonance

## Abstract

Atrial cardiomyopathy (ACM) represents a constantly evolving concept, with increasing importance in contemporary research and clinical practice. A better understanding of the mechanisms involved in atrial remodeling and its clinical correlations especially with atrial fibrillation (AF) and other cardiometabolic comorbidities may induce a significant impact on the diagnosis, prognosis, and therapeutic approach of ACM-related comorbidities. Although initially described several decades ago, investigators have only recently highlighted that several renal, metabolic, and cardiovascular diseases are determining factors for atrial remodeling and subsequent ACM. Based on data from multiple recent studies, our research emphasizes the correlations between ACM and other coexisting pathologies including cardiovascular, respiratory, or metabolic diseases, with fibrosis being the most incriminated pathophysiological mechanism. In addition to the usual tests, the paraclinical assessment of ACM is increasingly based on the use of various cardiac biomarkers, while the cardiac magnetic resonance (CMR) has become an increasingly tempting diagnostic too for describing morphofunctional aspects of the heart chambers, with the gadolinium contrast enhanced CMR (LGE-CMR) emerging as a commonly used technique aiming to identify and quantify the precise extent of atrial fibrosis. Further research should be conducted in order to clarify our knowledge regarding atrial remodeling and, therefore, to develop new and improved therapeutic approaches in these patients.

## 1. Introduction: Definitions and Classification

Atrial cardiomyopathy (ACM) and its clinical importance were first described in 1972 by Nagle et al. [1] in a study about a familial pathology concerning the atria, with first-degree heart block and supraventricular tachycardia degrading to a persistent atrial standstill. Recently, the European Heart Rhythm Association (EHRA), Heart Rhythm Society (HRS), Asia-Pacific Heart Rhythm Society (APHRS), and Sociedad Latinoamericana de Estimulación Cardiaca y Electrofisiología (SOLAECE) expert consensus proposed the following definition for ACM: “Any complex of structural, architectural, contractile, or electrophysiological changes affecting the atria with the potential to produce clinically-relevant manifestations [2]”.

The aim of this review is to depict some coordinates of ACM, from the molecular level to the therapeutic approach, for a better understanding of the mechanisms involved in atrial remodeling. Additionally, we reviewed some particular ACM clinical correlations, especially with atrial fibrillation (AF) and the common AF-related thromboembolic events, that may have an important impact concerning the diagnosis, prognosis, and therapeutic decision-making process in ACM.

High-incidence pathologies like chronic heart failure (CHF), AF, hypertension, diabetes mellitus (DM), and some other conditions including aging and obesity, are well-established causes of atrial remodeling. Although the induced changes share many similarities, there is an important histopathological substrate variability between the different clinical disorders [2,3]. Consequently, the EHRA histopathological classification was proposed for a better understanding of these differences that may influence the disease evolution and subsequent management. However, this descriptive classification does not provide information about the clinical severity or progression of the disease.

Class I: primarily cardiomyocyte changes (isolated AF, genetic diseases, and DM);

Class II: predominantly fibrotic changes (cigarette smokers and the elderly);

Class III: combined cardiomyocyte-pathology/fibrosis (CHF and valvular pathology);

Class IV: primarily non-collagen infiltration, with or without cardiomyocyte changes (accumulation of amyloid, fatty ACM, and inflammatory infiltrates) [2].

## 2. Pathogenesis of the Atrial Cardiomyopathy: Focus on Remodeling

There is an intimate relationship between atrial activity and ventricular systolic and diastolic performance; the main role of the left atrium (LA) is to manage the left ventricular filling pressures and cardiovascular performance by acting as a reservoir for the venous return during ventricular systole and as an additional pump for ventricular filling via an active atrial contraction [2,4,5].

Atrial remodeling is defined as a persistent change in the LA structure or function, possibly due to the appearance of atrial tachyarrhythmias such as AF and atrial flutter or secondary to pressure or volume overload related to CHF and other fibrotic disorders [6]. Furthermore, LA size and function have recognized importance in the prognosis and cardiovascular risk stratification in hypertensive, diabetic, and elderly patients [7].

Atrial remodeling is based on three principal pathophysiological pillars: structural, electrical, and functional remodeling.

Atrial structural remodeling is characterized by increased interstitial fibrosis and by maladaptive structural changes secondary to inflammation, pressure, or volume overload, leading to atrial enlargement [7,8]. One of the main components of cardiac remodeling is the progressive fibrotic tissue synthesis in the myocardium. Both the myocardial cells and the extracellular matrix (ECM), which consists predominantly of collagen fibers, can produce changes in the myocardial architecture caused by biochemical, mechanical, and electrical triggers [9,10,11]. While in healthy individuals the fibroblasts amount is relatively low, in certain pathological conditions their number increases significantly [9,12]. The proliferation of fibroblasts and the subsequent excessive production of ECM predisposes to the initiation and maintenance of electrical disturbances like anisotropy and re-entry formation [10,11].

Angiotensin II and transforming growth factor beta-1 are major stimulators of collagen production and key regulators of fibrosis. Angiotensin II and aldosterone are involved in inflammation and oxidative stress initiation. Recent studies have shown that inflammation and excess production of reactive species of oxygen seem to play a principal role in atrial remodeling [9,13]. Even a mild systemic inflammation can be associated with increased cardiovascular risk, especially since this proinflammatory status is found in many pathologies, such as hypertension, CHF, coronary artery disease, obesity, or DM. A number of studies indicate that oxidative reactions and chronic inflammation present in the conditions mentioned above can lead to atrial fibrillation; they are also present as a mechanism of endothelial dysfunction and arterial damage in hypertension [9,13].

Atrial functional remodeling is encountered in many pathological systemic processes and can cause alterations in atrial functioning regardless of the LA size [7,9]. This malfunction may be due to the abnormal propagation of electrical stimuli originated in various ectopic sources in the atria, decreased duration of action potential with multiple reentry circuits, and myocardial fibrosis that determines the heterogeneity of impulse conduction [9].

Electrical remodeling consists of a complex electrical substrate and ionic mechanism changes in the atria [14]. Factors that can modify the substrate are various and can affect the common electrophysiological pathway through the reduction of action potential duration, shortening the refractory periods, or altering the contractility mediated by calcium ions (Ca^2+^) and potassium fluxes from the myocytes [7]. The inadequate management of intracellular Ca^2+^ based on Ca^2+^ overload can affect cardiomyocyte excitation-coupling [12] promoting ectopic activity and apoptosis [15]. The most commonly diagnosed arrhythmias are supraventricular tachyarrhythmias, such as AF or atrial flutter [16]. Another contributor to the electrical remodeling of the atrium is the autonomic nervous system [15]. Autonomic ganglia are localized on the surface of the heart, predominantly in the pulmonary vein region; they adjust atrial electrical properties, thus playing an important role in arrhythmia initiation such as AF [2,17].

## 3. Etiology of Atrial Remodeling: New Concept, Classic Causes

**Figure 1:**
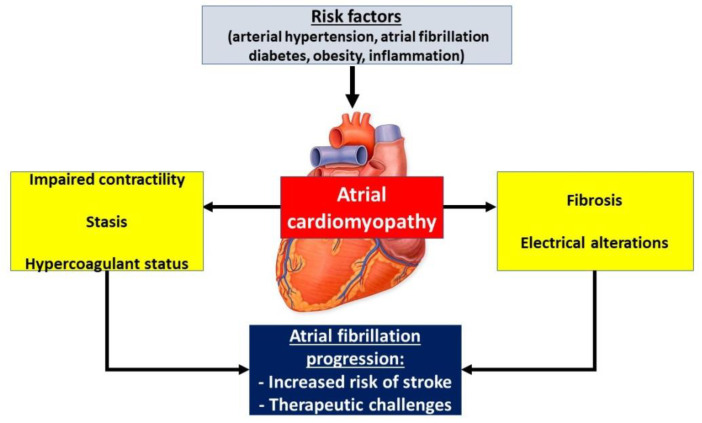
The main etiopathogenic mechanisms involved in atrial cardiomyopathy (adapted from Guichard et al.) [24].

Over the past decades, investigators have shown that many cardiovascular diseases such as high blood pressure [18], valvular disease [2], CHF [14,19], AF [20], or other various extracardiac conditions like obesity, diabetes [21], chronic kidney disease (CKD) [22], and obstructive sleep apnea (OSA) [23] are relevant risk factors for atrial remodeling. In Figure 1, we highlight the pathophysiological chain of events leading to the development and progression of ACM [24].

### 3.1. Arterial Hypertension

High blood pressure represents one of the main cardiovascular risk factors for ACM [7]. Previous studies have reported that in hypertensive animals, both atrial structural fibrotic changes and a significant decrease in the electrical conduction can be observed. Similar results were obtained by Medi et al. [18] who studied the electroanatomic and electrophysiological changes correlated to chronically treating systemic hypertension and left ventricular hypertrophy. The authors demonstrated an important reduction in global atrial conduction, functional conduction delay, and an increased predisposition for AF. These findings support a strong relationship between chronic high blood pressure and atrial or electrical remodeling. Maintaining optimal blood pressure values can promote reverse structural remodeling, showed by a significant decrease in volume of the LA [7].

Conditions highly associated with AF (especially hypertension) are often complicated by increased hemodynamic atrial load, leading to the overstretching of the atrial myofibriles. Available in vivo and in vitro data suggest that significant strain leads to hypertrophy, dedifferentiation, and ECM remodeling, as well as to electrical alterations, all these pathways being associated with an increased risk of AF and further atrial remodeling, thus closing a vicious pathophysiological circle [25].

### 3.2. Congestive Heart Failure

Normal function of the LA is necessary for optimal ventricular filling and adequate overall cardiac performance. Atrial dysfunction due to changes in the ventricular structure is a common finding in patients with CHF [2,14].

The LA volume is a sensitive indicator of LV filling pressure and reflects the degree of diastolic dysfunction, in the absence of severe valvular disease and/or AF. In order to reflect the LA volume’s capacity to reveal the chronicity of diastolic dysfunction, it has been proposed as the “glycosylated hemoglobin of diastolic dysfunction.” Also, because of divergent effects of preload and mural compliance on transmitral velocities, the mitral inflow patterns may appear normal (pseudonormal type of diastolic dysfunction) despite abnormal filling pressures. In such situations, the LA volume index (LAVI) provides the highest discriminative value in distinguishing between normal and pseudonormal transmitral filling patterns [26].

Why are these aspects important in patients with CHF? Mainly because the diastolic dysfunction represents the first phase of the so-called heart failure (HF) with preserved ejection fraction (EF > 50%). In these patients, a LA volume of ≥32 mL/m^2^ was an independent predictor of the first CHF episode [27].

A study conducted on 198 HF patients and 40 healthy controls compared the LA properties between HF with preserved and reduced ventricular ejection fraction. Investigators revealed that LA conduit, reservoir, and contraction functions are affected in diseased patients compared to control groups. Furthermore, they found that HF with preserved ejection fraction is associated with more dilatation and systolic dysfunction, while HF with reduced ejection fraction has an increased stiffness and susceptibility to AF [19]. In support of these findings, a recent study showed that HF was more common among patients with persistent AF than in those with the paroxysmal form [28].

### 3.3. Atrial Fibrillation

AF is the most frequent arrhythmia worldwide, affecting 25% of the adult population during their life [10]. AF occurs as a result of atrial remodeling induced by multiple cardiac and noncardiac diseases, and itself participates in the continuous development of remodeling causing the persistence of the arrhythmia [17]. Fibrosis is the underlying structural change involved in the pathogenesis of AF and in maintaining the myopathy state. Furthermore, atrial interstitial fibrosis generates changes in cellular coupling leading to a chronic substrate prone to anisotropy and re-entry. These findings support the concept that “AF begets AF” [15,29].

The pathways leading to thrombogenesis in AF are various, and there is evidence to suggest that all three elements of Virchow’s triad are present in AF: abnormal stasis of blood flow, endothelial dysfunction, and hypercoagulability [4].

The electrical and structural substrate within the LA in patients with paroxysmal and persistent AF has been studied by Teh et al. The investigators have demonstrated slower conduction, a higher proportion of decreased voltage, a lower global LA voltage, and an increased prevalence of complex signals in AF patients compared to the control group. These changes were more extensive in persistent AF [30].

Cardiac fibrosis consists of excessive deposition of collagen fibers in the ECM. High collagen accumulation has been revealed in LA tissue obtained from atriotomy in cadaveric patients with AF [29]. Stiles et al. [31] reported that patients with paroxysmal lone AF have an abnormal chronic bi-atrial substrate.

It is incompletely understood why some patients present repetitive episodes of paroxysmal AF for years or even for their entire lifetime, while other patients having identical comorbidities evolve into persistent AF in a rather short period of time. The above-mentioned concept of the negative “auto-amplification” that characterizes AF does not provide a detailed explanation for these differences. On the other hand, the results of atrial electroanatomic mapping in patients with AF and similar clinical findings were very different. These conclusions suggest the importance of a structural atrial condition defined as fibrotic atrial myopathy. However, even the presence of the most affected substrate can be shown in patients with paroxysmal AF instead of the persistent form [10,29].

An important link between AF and ACM is the inflammatory state [15]. The levels of interleukin-1 (IL-1) and interleukin-6 (IL-6), serum C-reactive protein (CRP), and tumor necrosis factor (TNF) have a predictive role in AF recurrence post-catheter ablation, and they are connected to the clinical progression of AF [9].

AF is a well-known risk factor for thromboembolic events such as ischemic stroke. In addition, atrial remodeling leads to a hypercoagulable state involved in thrombogenesis [10,15]. Specific conditions that enhance the thrombosis development in patients with AF include atrial dilation, atrial hypocontractility, endothelial injury, and increased secretion of pro-thrombotic factors including IL-6 and von Willebrand factor [32]. If in the case of hypercoagulant status, we have the therapeutic option of prophylaxis with various anticoagulants (though sometimes also limited by side-effects), it is important to remember the lack of adequate prophylactic agents against stroke that addresses atrial hypocontractility as a primary mechanism of atrial thrombosis.

Specific echocardiographic assessment of atrial wall velocities in AF patients revealed that there is a significant impairment of atrial contractility. Several hypotheses were raised concerning the link between atrial contractile dysfunction and the increased stroke risk:

- atrial hypocontractility persists for up to two months following AF conversion to sinus rhythm;
- atrial dilation and hypocontractility may aggravate despite catheter ablation of AF [4];
- atrial hypocontractility alone (without concomitant AF) is an independent risk factor for stroke [33].

### 3.4. Obstructive Sleep Apnea

Both OSA and LA enlargement are conditions that are commonly found in patients with cardiovascular diseases. Severe OSA may also increase LA size, independently of other risk factors. Several pathophysiological mechanisms induced by OSA have been suggested to explain why this condition may trigger or aggravate ACM. Increased sympathetic activity and systemic inflammation due to intermittent hypoxemia, increased intrathoracic pressure during the apneas are all causing supplementary atrial wall stress [34].

The basic mechanism by which OSA syndrome affects the atrial myocardial tissue is not completely understood. Dimitri et al. [23] investigated whether patients with this pathology present an abnormal atrial substrate and the potential relationship with recurrent fibrillation after cardioversion and catheter ablation. The study included 40 patients with moderate and severe forms of sleep apnea (diagnosed by polysomnography) which underwent ablation for paroxysmal AF. The authors revealed that patients with OSA have an increased atrial size, a reduction in atrial myocardial voltage, and prolonged conduction and sinus node recovery times. These findings support the assumption that atrial remodeling is a common condition in patients with OSA and an adequate therapeutic approach of the respiratory pathology may reverse (or at least slow-down) the progression of ACM.

### 3.5. Diabetes Mellitus

Deleterious effects of hyperglycemia in DM go beyond alterations in metabolic homeostasis, also modifying the vascular endothelium and inducing both direct and indirect myocardial injury. Increased inflammation and oxidative stress induce the formation of advanced glycation end products (AGEs), cellular apoptosis, mitochondrial dysfunction, and disorders of myocardial metabolism. An essential physiopathological step is the capacity of AGEs to infiltrate the myocardium leading to interstitial fibrosis and hypertrophy. All these events, reunited into the term DM cardiomyopathy, determine the substrate for anatomic and electrical atrial remodeling [35].

Additional negative prognosis factors are represented by an increasing prevalence of obesity, lack of regular physical activity, and exposure to stress, nowadays type 2 DM becoming one of the most common cardiovascular risk factors.

Over time, many studies have evaluated the influence of LA structural and functional remodeling in patients with type 2 diabetes by using various imaging methods. In patients with DM, echocardiography findings reveal that LA dysfunction can be a consequence of LA enlargement [21]. A recently published study on 431 patients with type 2 DM and no history of cardiovascular disease demonstrated that increased LAVI is accompanied by major cardiovascular events and decreased life expectancy [36].

### 3.6. Obesity

Although many studies have shown that obese individuals have LA enlargement, given that the atrial size is typically quantified by indexing it to height or body surface, the final figures are relatively similar to non-obese controls. However, given the increased prevalence of obesity-associated comorbidities which can also influence cardiovascular remodeling, the real, independent impact of obesity on ACM can be difficult to assess [37].

Epicardial fat is metabolically active and releases inflammatory cytokines, adipokines, and free fatty acids that are involved in the progression of atrial fibrotic remodeling. As a result, local epicardial adiposity can promote the substrate for arrhythmias. There is also a strong relationship between the quantity of pericardial fat and AF prognosis. Lifestyle changes, weight loss, normal blood pressure, and optimal glycemic values were connected with a better rhythm control among obese patients with AF after catheter ablation [10].

### 3.7. Chronic Kidney Disease

The prevalence of AF increases with age in line with a decline in the estimated glomerular filtration rate (eGFR). Various reports claim that 7–18% of patients with CKD had AF, while 10–33% of patients were incidentally found with CKD at the time of the newly diagnosed AF. The occurrence of AF may promote the progression of CKD and increase the risk of developing end-stage renal disease. Moreover, the coexistence of AF and CKD (eGFR < 60 mL/min/1.73 m^2^) was related to adverse cardiovascular events and all-cause mortality [38].

CKD is associated with enhanced activation of the renin-angiotensin-aldosterone system and excessive sympathetic excitation, which can further promote atrial remodeling. Furthermore, dysfunctions of the autonomous nervous system activity might trigger different signaling pathways, including proinflammatory cytokines, epicardial adipose tissue, and oxidative stress, which could also promote ACM [39]. Additionally, patients with CKD are characterized by a marked pro-thrombotic status, which indicates an increased synthesis of thrombogen molecules, doubled by endothelial dysfunction, subclinical inflammation, elevated levels of plasminogen activator inhibitor-1, and abnormal coagulation factor activities [38].

Sciacqua et al. [22] investigated the relationship between CKD and LAVI in predicting the occurrence of AF. The study was performed on 3549 subjects without preexistent AF and thyroid conditions. The authors revealed that increased LAVI has a role in AF prediction, even in the absence of renal dysfunction. Another important finding was that LAVI and renal impairment have a synergic effect on the prognosis of heart rhythm disorders, independent of other cardiovascular risk factors. Investigators have also demonstrated that the risk of AF was twice as high as expected in the presence of left ventricular hypertrophy and renal disease.

## 4. Clinical Overview in Patients with Atrial Cardiomyopathy: From Fibrosis to Thrombosis

ACM can often be considered a substrate of AF, its involvement in the occurrence of thromboembolic events is well documented [15].

Numerous studies have demonstrated that the association between AF, inflammation, atrial fibrosis, and thromboembolism has important clinical implications [10]. As mentioned, AF promotes the release of IL-6 and interleukin-8, TNF-α, and angiotensin II, which are key-factors in many cardiovascular pathologies. Angiotensin II and endothelin-1 have a well-known role in promoting myocardial inflammation and susceptibility to arrhythmias. Even if systemic inflammation supports the induction and persistence of fibrillation, it can also contribute to thrombus formation with subsequent thromboembolic events [2].

Although the stroke represents the most severe complication of AF, its underlying mechanisms are much more complex than only a simple cardiogenic embolism. In-depth studies have shown that endothelial dysfunction, various procoagulant molecules due to inflammation, impaired contractility of LA, and stasis are all determining a plethora of structural changes in the myocardium, outlining a persistent thrombogenic substrate [10].

AF is the most common risk factor for thromboembolism whether the arrhythmia is long-standing or not. However, recent findings suggested that the embolic risk is higher in patients with persistent or permanent AF than in those presenting with the paroxysmal form [15]. Interestingly, the ASSERT trial proved that there is no temporal relation between systemic embolism and the occurrence of AF embolic events [40].

**Figure 2:**
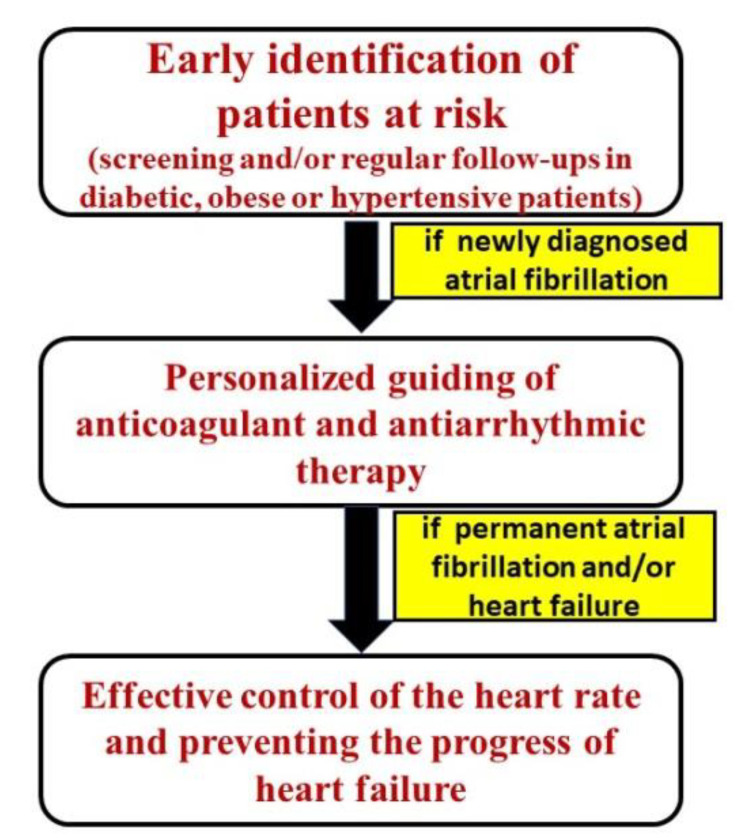
Algorithm for the clinical approach in patients with suspected ACM [24].

The concept of ACM can serve in a practical manner for the clinical approach of patients with certain cardiometabolic pathologies (e.g., diabetes, arterial hypertension, dyslipidemia, etc.). A proposed algorithm is found in Figure 2 [24].

## 5. Paraclinical Investigations for Atrial Cardiomyopathy: A State-of-the-Art Approach

According to current literature data, the dosing of novel biomarkers, as well as imaging explorations aimed to quantify and identify fibrosis, can be effective for the diagnosis of atrial remodeling.

### 5.1. Biomarkers

Multiple studies have demonstrated that high blood levels of CRP and IL-6 are associated with an increased incidence of AF, both among patients undergoing cardiac surgery and the general population. Increased levels of the above-mentioned inflammatory biomarkers are also correlated with a significant risk of arrhythmia recurrence in patients who underwent electrical cardioversion or catheter ablation. Further supporting these findings, a recent study showed a decline in CRP levels after the conversion to sinus rhythm. Very interesting, the duration of AF does not influence the level of these markers [2,13].

IL-6 can also interfere with the pro-thrombotic state during AF, by endothelial activation and platelet aggregation [4,10]. Increased levels of fibrinogen and TNF-α are also related to the persistence of AF and, therefore, to a continuum of atrial remodeling [13].

An increased level of N-terminal pro-B-type natriuretic peptide (NT-proBNP) represents an established hallmark in patients with HF, however, this biomarker is markedly influenced by the presence of concomitant AF, making it difficult to distinguish between HF versus AF as the primary determinant of a raised NT-proBNP. To shed some light on this controversy, a very recent study compared the plasma levels of NT-proBNP in patients with HF in AF versus HF in sinus rhythm and found that baseline NT-proBNP serum concentrations of patients who had AF were higher than those of patients in sinus rhythm, regardless of whether the latter had a history of AF or not [41]. Moreover, the same study revealed that a novel biomarker for HF-growth differentiation factor-15 (GDF-15) is not influenced by the presence of AF. Based on these findings, a multimarker approach with the assessment of GDF-15 and NT-proBNP could provide additive value in the initial evaluation of patients with HF and concomitant AF. Additionally, GDF-15 may predict the thromboembolic risk in patients with AF [10].

Atrial changes induced by AF, such as increased atrial size and fibrosis, stimulates the secretion of natriuretic peptides. Atrial natriuretic peptide (ANP) is a cardiac hormone with vasodilatory, antihypertrophic, diuretic, and natriuretic effects. Therefore, ANP is involved in maintaining the water-sodium balance and optimal blood pressure. A previous study revealed that mice with ANP deficiency developed hypertension and cardiac hypertrophy as a result of ischemic remodeling and pressure overload. More recently, ANP has been shown to have pro-angiogenetic, anti-inflammatory, and anti-atherosclerotic properties, being consequently involved in the underlying mechanisms of cardiac remodeling [42,43].

Given the increased prevalence of AF and atrial remodeling among patients with HF, several studies aimed to evaluate the utility of HF biomarkers in detecting ACM. Denysiak et al. proved that soluble interleukin 1 receptor-like 1 (ST2) is a marker of advanced functional remodeling in patients with AF, and is associated with significantly longer ablation procedures independent of LA size [44], while other authors reported that increased LA volumes are correlated with higher serum ST2, marked fibrosis and poor outcome in patients with HF [45].

Another promising biomarker of cardiac fibrosis is represented by syndecan-1, especially in patients with HF with preserved ejection fraction (EF). Given the predominance of diastolic dysfunction in these patients (due to myocardial hypertrophy and subsequent fibrosis), an early assessment of the myocardial fibrotic injury is paramount in determining the chances for cardioversion or, if not the case, of maintaining an adequate heart rate [46].

### 5.2. Imaging Techniques

Numerous imaging explorations are used currently in clinical practice to identify LA remodeling [7]. Echocardiography (two-dimensional images, pulsed-wave Doppler, two-dimensional speckle-tracking echocardiography, strain and strain rate imaging), doubled by a cardiac computed tomography, provide extensive information on atrial volumes and function [10]. Although alterations of LA function may occur before the volume changes of the LA, an increase in LA volume > 15% can be suggestive of atrial remodeling [7].

Some new techniques, like the evaluation of the LA volume by 3D-Echocardiography, represent more accurate methods than the classic determination of the LA size. Two-dimensional speckle-tracking echo is a more sensitive imaging method for the early assessment of functional remodeling, even before the appearance of visible anatomical changes in biopsies [2]. Global LA strain imaging is superior to conventional volumetric measures in predicting cardiovascular diseases and metabolic disorders. By estimating spatial gradients in myocardial velocities, strain and strain rate imaging can provide information about myocardial remodeling [1,21].

Cardiac computed tomography is superior to 2D-Echocardiography in the assessment of atrial volumes, and it can be used in thrombus screening before undergoing AF ablation [2]. Recently, cardiac magnetic resonance (CMR) has become the gold standard for describing heart chamber structure and function. Late-gadolinium-enhanced CMR (LGE-CMR) has been used to identify and quantify the extent of atrial fibrosis. The amount detected by LGE-CMR was correlated with the risk of post-ablation recurrent AF. This imaging technique may guide the selection of patients who may benefit from ablation. Recent studies used LGE-CMR for the characterization of scar tissue formation after the ablation procedure because an incomplete scar, with gap lesions, is associated with an increased risk of recurrences due to incomplete electrical isolation. Another aspect supporting this finding is that the ablation lesion burden was correlated, on LGE scans, with the low-voltage areas on electro-anatomical mapping systems [15,47].

In the light of some latest research, the assessment of epicardial adipose tissue (EAT) depots in cardiovascular pathology is of particular interest. Various studies that successfully used CMR for the evaluation of EAT volume and thickness found interesting correlations between the presence of fatty tissue and the atrial remodeling, highlighting areas of low voltage, conduction impairment, and greater fractionation of electrograms in anatomical regions adjacent to significant epicardial fat depots [48]. A study that used a high-performance, improved CMR, with a 3-dimensional multi-echo Dixon fat-water separated sequence, revealed that AF patients had significantly larger LA-epicardial fat (28.9 ± 12.3 and 14.2 ± 7.3 mL for AF and non-AF, respectively; *p* < 0.001), even after adjusting it to LA volume. Moreover, combined evaluation of LA EAT and LA volume provided additive diagnostic value and superior performance in the detection of AF compared to the assessment of online LA volume (*p* < 0.001) [49]. Very interesting, albeit atrial remodeling is very common in dilated cardiomyopathy (DCM), the load of EAT (assessed by CMR) is decreased in these patients compared to healthy controls, irrespective of LV and/or RV systolic dysfunction. In contrast, if only assessing the patients with DCM, an increase in LV mass and volumes was associated with a significantly greater amount of EAT [50].

Even if CMR is considered the gold standard for visceral adipose tissue assessment, the majority of clinical studies that assessed EAT to date were performed using computed tomography (CT), because of its adequate spatial resolution, excellent reproducibility, and ease to perform. Additionally, in several situations, EAT was quantified on a CT scan performed for other pathologies [51]. The results obtained by this method were similar to CMR findings: CT-assessed EAT was independently associated with both increased prevalence of AF [52,53] and other indirect markers for atrial remodeling, such as increased LV mass, LV end-diastolic diameter, or LV end-diastolic volume [54].

A novel noninvasive method used for the assessment of atrial flow dynamics is the 4-dimensional (4D) flow CMR. This method is more accurate than the trans-esophageal echocardiography and provides a comprehensive detection and quantification of stasis in the LA and LA appendage (the most prone location for thrombus formation) [15].

Electroanatomic mapping is the standard invasive method in ACM substrate characterization, through the geographic display of signal amplitude data and by rendering the atrial surfaces. It can be used to investigate atrial myopathy associated with sinus node disease, rheumatic mitral stenosis, atrial septal defects, CHF, OSA syndrome, and aging [2].

## 6. Particular Therapeutic Approaches

### 6.1. Pharmacological Treatment

Cardiac remodeling includes functional, electrical, and structural changes that are closely interconnected, and influence both the therapeutic approach and the prognosis. The practical management of ACM is based on stroke prevention, cardiac rhythm therapy, rate control, and ablation strategy [24].

Amiodarone is the most effective and frequently used antiarrhythmic drug. However, recent recommendations emphasize catheter ablation rather than amiodarone for AF treatment [14]. Previous studies demonstrated that the outcome of AF ablation depends on the extent of atrial fibrosis. Therefore, the evaluation of fibrosis may be important in making the therapeutic decision, as we already mentioned above.

The importance of metabolic effects on ACM is supported by the fact that weight loss and risk factor management induce better post-ablation results and a decreased rate of AF recurrence [3,24]. However, in patients with recurrent AF after pulmonary vein ablation, statin therapy had no beneficial effects. Therapy with angiotensin-converting enzyme inhibitors (ACEI) and angiotensin II receptor blockers has shown promising results in reducing the incidence of AF in patients with a history of cardiovascular disease and in those undergoing cardiac surgery and treated with statins. In addition, ACEI therapy had positive results when co-administered with spironolactone and statins [3].

Furthermore, other therapies have been shown to be useful in atrial remodeling. ANP promotes natriuresis, diuresis, vasodilation, and inhibits aldosterone and renin secretion. Given its effects, ANP is considered a promising drug for various cardiovascular diseases. It has been proven that ANP administration reduces maladaptive cardiac remodeling and has anti-inflammatory and proangiogenic effects. The inhibition of neprilysin results in an increased ANP secretion. Therefore, the inhibition of neprilysin and angiotensin receptors is a novel therapy in patients with HF [43]. Thiazolidinedione, used in the treatment of type 2 DM, exhibits anti-inflammatory and antioxidant properties, thus having the potential to improve cardiac remodeling [10].

Another potential therapeutic target in improving atrial contractility is the Ca^2+^- sensitizing agent levosimendan. This option is sustained by some evidence concerning dysregulation of the myofilament response to Ca^2+^ release or Ca^2+^ impaired sensitivity, which are significant contributors to contractile dysfunction in AF [4]. Intracellular Ca^2+^ homeostasis and subsequent contractility efficiency rely upon phosphorylation processes, which, in AF, are pathologically altered. Some recent studies have shown that levosimendan increases cerebral blood flow, decreases NT-proBNP (an indirect marker of adverse atrial remodeling), and improves atrial pump function on echocardiography; however, it also increases the occurrence of AF in these patients, an undesired effect attenuated by concomitant treatment with ranolazine, an inhibitor of the late inward sodium current [55].

Some very recent reports were focused on unravelling the pleiotropic, cardiovascular protective effects of sodium–glucose cotransporter-2 (SGLT2) inhibitors, a novel class of oral hypoglycemic medication that showed very promising results in the treatment of HF. A study conducted by Shao et al. revealed that empaglifozin, a SGLT2 inhibitor, can prevent atrial structural and electrical remodeling as well as ameliorate mitochondrial metabolism in diabetic rats, via the peroxisome proliferator-activated receptor-c coactivator 1α (PGC-1α)/nuclear respiratory factor-1 (NRF-1)/mitochondrial transcription factor A (Tfam) signaling pathway [56]. Moreover, the same authors highlighted that empaglifozin not only attenuated DM-related structural alterations (atrial interstitial fibrosis, atrial myocyte hypertrophy) but also determined anti-inflammatory effects (by decreasing C-reactive protein) and prevented AF inducibility during electrophysiological studies.

Previous studies have drawn an interesting new direction concerning the possible roles of oxidative stress and inflammation in the mechanisms of the electrical and structural substrates for atrial remodeling and subsequently, AF [57,58]. Even a transient surge in the oxidative stress can induce atrial interstitial fibrosis, promoting not just structural remodeling, but also electrical instability with a consecutive increase in AF inducibility [59].

It was demonstrated that another SGLT2 inhibitor, canaglifozin, exhibited significant antioxidant properties in the atrial tissue. This effect was related to increased ketogenesis promoted by canaglifozin, thus enhancing the myocardial uptake of ketoacids (e.g., acetoacetic acid), which competes with fatty acid oxidation. Cardioprotection is based exactly on this metabolic switch, the important metabolic efficiency of ketone body oxidation being well-established in the literature [60]. Given the major involvement of reactive oxygen species in the pathogenesis of AF, a recent study further confirmed the antioxidant effects of canaglifozin and its atrial anti-remodeling capacity, by demonstrating that this SGLT2 inhibitor suppressed an increase of AF inducibility [59].

### 6.2. Invasive Approach

Cardiac resynchronization therapy (CRT) has been shown to improve not only the LV ejection fraction but also LA function. A study including a total of 41 patients who received a de novo CRT implantation revealed that the P-wave duration measured in the surface 12-lead ECG significantly decreased following CRT therapy (at least 12 months since implantation and with adequate bi-ventricular pacing > 92% of the time). This is an important clue regarding the therapeutic potential use of CRT in patients with ACM because the prolongation of the P-wave duration represents a quantitative indirect marker for atrial electrical remodeling [61].

CRT’s capacity to induce LV and LA reverse remodeling might be partly related to the improved LV filling and subsequent increase in LV contractility, cardiac output, and LV ejection fraction. In a similar manner, ameliorated diastolic function and optimal LA filling are also enhancing LV contractility. Furthermore, synchronous triggering of ventricular depolarization also decreases mitral regurgitation, thus contributing to LA reverse remodeling [62,63,64].

The results from a MADIT-CRT (Multicenter Automatic Defibrillator Implantation Trial with Cardiac Resynchronization Therapy) study comprising 533 patients with CRT and defibrillator (CRT-D) showed that >40% of the patients presented complete left-sided reverse remodeling (above-median change in both LA volume and LV end-systolic volume, this aspect being associated with a significantly lower risk of HF and death [64].

Even if they are not a therapeutic option per se in ACM, left ventricular assist devices (LVADs) and their increasing availability in patients with end-stage HF play an important role in the prognosis of patients with impaired atrial function. Commonly used either as a bridge therapy to heart transplantation or following precise, limited indications, LVADs can be an arrhythmogenic trigger, as was shown in many studies that highlighted the frequency of AF occurrence in both pre-LVAD or post-LVAD patients with advanced HF, with an estimated prevalence of 21–54% and a great impact upon patients’ outcome and mortality rates [65,66]. Moreover, LVAD support means that left ventricular filling is not compromised by the loss of atrial contraction due to AF; in contrast, RV filling and consequently cardiac output are still impaired by the loss of atrial contraction, especially in patients with previous RV dysfunction and pulmonary hypertension. However, taken into account the plethora of mechanisms involved in the atrial injury in patients with severe HF needing LVADs, the precise role of this invasive approach in ACN needs to be furtherly ascertained.

Under these circumstances, it was hypothesized that LVAD implantation may have a significant impact upon atrial remodeling through modifications in atrial filling pressures and myocardial strain. A recent study that evaluated several echocardiographic atrial parameters before and after LVAD implantation pointed out that LA size and volume index were significantly decreased after LVAD implantation, and a significant proportion of patients with previous paroxysmal AF presented no further documented AF episodes. Nevertheless, the occurrence of a post-implantation, new-onset AF was associated with poor outcome and increased mortality in patients receiving LVAD. Basically, these latest insights suggest that LVAD induces reverse atrial electrical and structural remodeling, but arrhythmic events may still severely influence the clinical outcome of patients with LVAD [67].

Even if the topic is too vast to be exhaustively covered by this review, we need to mention the anti-remodeling role of catheter ablation for isolated AF which was confirmed in multiple studies during the last two decades [68,69,70]. This reverse remodeling capacity is primarily associated with the maintaining of the sinus rhythm after ablation, with significant reductions in both LA diameters and volumes. For example, the left atrial dimensions at three months (44  ±  6 versus 40  ±  6 mm, *p* < 0.001) and six months (44  ±  6 versus 40  ±  6 mm, *p* < 0.001) were significantly smaller compared with just after ablation in the patients without AF recurrence. Additionally, the authors found a significant decrease of the natriuretic peptide concentrations between baseline levels and those at three and six months (111.0 versus 34.9 pg/mL, *p*  <  0.001; 111.0 versus 30.4 pg/mL, *p*  <  0.001), thus furtherly confirming the potential benefits of catheter ablation not just for AF management, but also for the outcome of patients with ACM and HF [71].

### 6.3. Computational Models

In recent years, novel computational models and applications for atrial remodeling entered the spotlight, thus offering a framework for a better approach to AF ablation, pharmacotherapy, and the subsequent long-term prognosis of these patients [72].

Basically, these complex, multi-scale atrial models are mathematical models that are bridging the molecular, cell-scale mechanisms with the tissue alteration and finally, with the whole atria scale dysfunction, integrating this pathophysiological continuum into a graphical representation of atrial electrophysiology [73].

The cell scale provides information concerning the kinetics of different ionic channels and the coupling of some regulatory proteins in order to produce the transmembrane potential of the atrial myocytes, while the tissue scale highlights the intercellular coupling and fiber orientation that exerts a great influence upon electrical propagation. Finally, the whole atria scale of these computational models provides insights into the segmentary distribution of normal tissue or fibrotic areas, also generating a 3D reconstruction of the atrial geometry that simulates atrial activation [72].

The clinical utility of computational relies on their capacity to allow the study of some drugs’ effects on a wide range of cellular phenotypes, thus providing an improved prognosis tool concerning the impact of certain substances on the ionic currents. For example, recent research emphasizes the development of multi-target therapies that aim to combine the efficacy of Na^+^ channel blockade with repolarization modulating drugs to generate ideal compound profiles that could reverse atrial remodeling or at least inhibit its underlying electro-structural mechanisms [74].

Another important aspect with direct clinical impact refers to the pharmacokinetic (PK) and pharmacodynamic (PD) modeling of some antiarrhythmic substances such as drug conversion from amiodarone to its derivative dronedarone. Both substances exhibit a similar main structure (removed iodine and added methanesulfonyl group) and overall electropharmacologic profile but with different effects on individual ion channels. Moreover, concerning PK models, amiodarone has increased affinity to accumulate in different tissues due to a longer half-life, thus raising the risk for non-cardiac side-effects. In this context, PK and PD modeling should be routinely considered for drug development, providing a better understanding of several dynamic drug–ion channel interactions involved in ACM and their subsequent positive or negative (non)cardiac outcomes [75].

## 7. Conclusions

ACM represents a complex entity that is correlated with numerous commonly met conditions from clinical practice, including systemic hypertension, congestive HF, AF, type 2 diabetes, sleep apnea syndrome, and obesity. Fibrosis is the most important pathophysiological mechanism of atrial remodeling, its accurate assessment representing the foundation for an adequate therapeutic approach and an improved long-term prognosis.

Besides the classic inflammatory biomarkers or natriuretic peptides, laboratory diagnosis of ACM is focused on modern biomarkers that could indicate the magnitude of the fibrogenic process and also assess the risk of future cardiovascular events. CMR has become a promising and increasingly used technique for describing heart chamber structure and function, LGE-CMR being commonly used to assess the extent of atrial fibrosis and offering an accurate characterization of scar tissue formation after the ablation procedure and thus ascertaining the need for an eventual reintervention.

The ultimate goal will be to translate recent molecular and clinical findings into an algorithm-based therapy for patients diagnosed with conditions suggestive of ACM. In addition to classical pharmacological drugs used in HF or AF, invasive devices (e.g., CRT-D) or the use of computational models may be a viable option for both the acute and long-term therapeutic optimization of patients with HF and ACM.
